# Prooxidant Mechanisms in Toxicology

**DOI:** 10.1155/2014/308625

**Published:** 2014-03-20

**Authors:** Afaf K. El-Ansary, Malak Kotb, Maha Zaki Rizk, Nikhat J. Siddiqi

**Affiliations:** ^1^Biochemistry Department, College of Science, King Saud University, P.O. Box 22452, Riyadh 11495, Saudi Arabia; ^2^Department of Molecular Genetics, Biochemistry and Microbiology, University of Cincinnati College of Medicine, Cincinnati, OH, USA; ^3^Therapeutic Chemistry Department, National Research Center, Dokki, Cairo, Egypt

Oxidative stress is an important aspect of toxicology and therefore of considerable interest. It can be traced back to 1954 when Gerschman et al. propounded the free radical theory which for the first time implicated partially reduced forms of oxygen in its toxic mechanism [1]. This was followed by Harman in 1956 proposing the concept of free radicals playing a role in the ageing process [2]. The discovery of superoxide dismutase by McCord and Fridovich in 1969 [3] was a second landmark in the role of free radicals in biological systems [4]. The third era of free radicals in biological systems dates back to 1977 when Mittal and Murad [5] provided evidence that the hydroxyl radicals, ^•^OH, stimulate activation of guanylate cyclase and formation of the “second messenger” cyclic guanosine monophosphate [4]. Since then a large body of evidence indicates that living systems not only generate free radicals but also have developed mechanisms for simultaneous coexistence and optimal use of free radicals to their advantage. The cellular defenses include low molecular weight free radical scavengers such as reduced glutathione, *α*-tocopherol, thioredoxins, and ascorbic acid, as well as enzymatic defenses such as superoxide dismutase, catalase, and glutathione peroxidase. Biological sources of free radicals include mitochondrial electron transport chain, enzymes like cytochrome P450, xanthine oxidase, and phagocytosis. Reactive oxygen species (ROS) and reactive nitrogen species (RNS) are well known for playing a dual role as both deleterious and beneficial species, since they can be either harmful or beneficial to living systems [4]. Beneficial effects of ROS occuring at low/moderate concentrations involve physiological roles in cellular responses to anoxia, in defense against infectious agents and in the function of a number of cellular signaling systems. One further beneficial example of ROS at low/moderate concentrations is the induction of a mitogenic response [4]. The harmful effect of free radicals causing potential biological damage is termed oxidative stress and nitrosative stress [4]. This occurs in biological systems when there is an overproduction of ROS/RNS on one hand and a deficiency of enzymatic and nonenzymatic antioxidants on the other hand. Therefore oxidative stress represents a disturbance in the equilibrium status of prooxidant/antioxidant reactions in living organisms. Excess of ROS can damage cellular lipids, proteins, nucleic acids, and other macromolecules inhibiting their normal functions.

In addition to disease states, oxidative stress has been implicated in the mechanisms of drug-induced toxicity [6], chemical toxicity [7], and more recently in nanoparticles-induced toxicity. Metals like iron, copper, chromium, vanadium, and cobalt undergo redox-cycling reactions and thus participate in free radical generation. Other metals like mercury, cadmium, and nickel are toxic by their ability to deplete glutathione and bind to sulfhydryl groups of proteins [8]. Toxicants cause tissue damage via diverse mechanisms, many of which involve activation of cell survival and apoptotic pathways. One of the key areas of recent interest is the role that oxidative stress and nitrative stress play in mediating the response to toxicants via this cytotoxic pathway [9]. However the question whether uncontrolled formation of ROS is a primary cause or a downstream consequence of the pathological process remains to be answered [4]. Therefore in this special issue an attempt has been made to include reviews and research papers which update our knowledge about the role of free radicals in the toxicological processes and identify gaps in knowledge which would lead to a better understanding of toxicological pathogenesis.

*Afaf K. El-Ansary* *Malak Kotb* *Maha Zaki Rizk* *Nikhat J. Siddiqi*

## References

[1] Gerschman R, Gilbert DL, Nye SW, Dwyer P, Fenn WO (1954). Oxygen poisoning and X-irradiation: a mechanism in common. *Science*.

[2] Harman D (1956). Aging: a theory based on free radical and radiation chemistry. *Journal of Gerontology*.

[3] McCord JM, Fridovich I (1969). Superoxide dismutase. An enzymic function for erythrocuprein (hemocuprein). *Journal of Biological Chemistry*.

[4] Valko M, Leibfritz D, Moncol J, Cronin MTD, Mazur M, Telser J (2007). Free radicals and antioxidants in normal physiological functions and human disease. *International Journal of Biochemistry and Cell Biology*.

[5] Mittal CK, Murad F (1977). Activation of guanylate cyclase by superoxide dismutase and hydroxyl radical: a physiological regulator of guanosine 3′,5′-monophosphate formation. *Proceedings of the National Academy of Sciences of the United States of America*.

[6] Deavall DG, Martin EA, Horner JM, Roberts R (2012). Drug-induced oxidative stress and toxicity. *Journal of Toxicology*.

[7] Rashid K, Sinha K, Sil PC (2013). An update on oxidative stress-mediated organ pathophysiology. *Food and Chemical Toxicology*.

[8] Valko M, Morris H, Cronin MTD (2005). Metals, toxicity and oxidative stress. *Current Medicinal Chemistry*.

[9] Roberts RA, Laskin DL, Smith CV (2009). Nitrative and oxidative stress in toxicology and disease. *Toxicological Sciences*.

